# Aquatic Toxicity of Antibiotics Ciprofloxacin and Sulfamethoxazole: Significant Impact on Early Life Stages of Crustaceans, a Marine Diatom, and a Freshwater Plant

**DOI:** 10.3390/toxics13110979

**Published:** 2025-11-14

**Authors:** Edoardo Pietropoli, Rebecca Zgheib, Marco Selmo, Giacomo Melotto, Rosa Maria Lopparelli, Lorena Lucatello, Marianna Pauletto, Marco De Liguoro

**Affiliations:** Department of Comparative Biomedicine and Food Science (BCA), University of Padova, 35020 Legnaro, Italy; edoardo.pietropoli@unipd.it (E.P.); rebecca.zgheib@phd.unipd.it (R.Z.); rosa.lopparelli@unipd.it (R.M.L.); lorena.lucatello@unipd.it (L.L.); marco.deliguoro@unipd.it (M.D.L.)

**Keywords:** ciprofloxacin, sulfamethoxazole, aquatic ecotoxicology, environmental risk assessment

## Abstract

The occurrence of antibiotic residues in the environment is of concern not only because of their contribution to the spread of bacterial resistance, but also due to their possible toxicity to non-target organisms. In this study, the aquatic environmental toxicity of ciprofloxacin (CIP) and sulfamethoxazole (SMX) was assessed in the following model organisms: *Daphnia magna* and *Artemia salina* (embryonic and immobilisation test with a 10-d follow-up), *Phaeodactylum tricornutum* (algal growth inhibition test), and *Spirodela polyrhiza* (duckweed growth inhibition test). Results showed that among the two saltwater organisms, *A. salina* was insensitive to both antibiotics, whilst *P. tricornutum* responded only to SMX with an EC_50_ of 2.7 mg L^−1^. In freshwater species, *D. magna* embryos were more sensitive than juveniles to SMX (EC_50_ 53.8 and 439.2 mg L^−1^, respectively), whereas the opposite trend was observed for CIP (EC_50_ 95.9 and 15 mg L^−1^, respectively). *S. polyrhiza* confirmed the remarkable sensitivity of aquatic plants to fluoroquinolones, with EC_50_ values between 0.28 and 0.34 mg L^−1^ depending on the endpoint considered. Notably, this species was also more sensitive to SMX than expected, with EC_50_ values between 1.5 and 2.5 mg L^−1^, which are an order of magnitude lower than those typically obtained with *Lemna* spp. exposed to sulphonamides. Considering the high environmental input of these antibiotics from both human and veterinary treatments, adverse effects on aquatic plants cannot be excluded, potentially leading to ecosystem-level consequences.

## 1. Introduction

Pharmaceuticals, whilst essential for human and animal health, can have unintended consequences on the environment. After administration, many drugs are excreted unchanged or as active metabolites, entering sewer systems and reaching wastewater treatment plants that are often unable to remove them completely. Unused medications are also improperly disposed of, contributing to environmental contamination. Intensive livestock farming is another major source of pharmaceutical pollution. Antibiotics, in particular, are frequently used for mass medication and considerable quantities can be released into the environment through manure application and agricultural runoff [1]. Once in natural ecosystems, these bioactive substances can interact with non-target organisms, potentially interfering with essential cellular functions and ecological processes. The presence of antibiotics also raises concerns about the spread of antimicrobial resistance, which poses risks to both environmental and public health.

The fluoroquinolone ciprofloxacin (CIP) and the sulphonamide sulfamethoxazole (SMX) are two synthetic antibiotics that have been widely used in human medicine for decades. Due to their relative resistance to degradation [2,3], both compounds are frequently detected in various environmental matrices [4]. Their environmental occurrence is also associated with farming activities. In fact, CIP is the main metabolite of enrofloxacin, commonly used in animal husbandry [5,6], and SMX itself, typically in combination with trimethoprim, is also widely employed. Their veterinary usage is mainly in the form of metaphylactic mass-medication, which implies a relevant environmental drug load and involves various farm settings, including aquaculture [7,8]. Additionally, effluents from pharmaceutical manufacturing facilities have been identified as significant point sources of CIP and SMX contamination in some regions [9,10]. According to a review by Kovalakova et al. [11], the average concentrations of CIP and SMX in surface water are around 10^3^ and 10^2^ ng L^−1^, respectively, with peak values reaching 10^7^ and 10^4^ ng L^−1^.

Both CIP and SMX act by inhibiting bacterial DNA synthesis, albeit through distinct molecular mechanisms. However, their limited selectivity can result in adverse effects on non-target organisms [12,13]. Previous ecotoxicity studies conducted by our research group have highlighted intriguing toxicological profiles for other fluoroquinolones and sulphonamides. For example, the fluoroquinolones enrofloxacin, flumequine, and levofloxacin induced embryotoxicity and delayed acute toxicity after neonatal exposure in *Daphnia magna* [14]. Moreover, chronic exposure to flumequine leads to transgenerational toxicity in this species [15], with several genes related to growth, development, structural components, and antioxidant response significantly modulated [16]. On the other hand, the sulphonamide sulfamethazine, although showing low acute toxicity (EC_50_ = 202 mg L^−1^), exhibited pronounced toxicity after a reproduction inhibition test in *D. magna*, with an estimated EC_50_ of 4.25 mg L^−1^; notably, normal reproduction cycles were disrupted, with frequent egg abortion [17]. When it comes to primary producers, fluoroquinolones have demonstrated high toxicity to aquatic plants, with EC_50_ values ranging from 0.05 to 2.5 mg L^−1^ [12]. In contrast, sulfonamides tend to be more toxic to algae, showing EC_50_ values between 0.2 and 43 mg L^−1^ [18,19].

Given this background, the present study aims to assess the embryonic and neonatal toxicity of CIP and SMX in two crustacean species (*D. magna* and *Artemia salina*), as well as their growth inhibition potential in the freshwater duckweed *Spirodela polyrhiza* and the marine diatom *Phaeodactylum tricornutum*. To evaluate potential delayed acute effects, toxicity tests on neonate crustaceans included an extended post-exposure observation period of 10 days in clean medium [14]. Furthermore, to increase the sensitivity of the growth inhibition test on *S. polyrhiza*, various endpoints in addition to frond number were considered, including frond weight, root growth, total frond area, and frond greenness. Beyond comparing sensitivity across taxa and life stages, an additional aim of the present study was to conduct a screening-level risk assessment by deriving provisional freshwater PNEC (Predicted No Effect Concentration) from the most sensitive endpoint and comparing these values and PNEC previously obtained from Species Sensitivity Distribution (SSD) with concentrations reported in environmental monitoring.

## 2. Materials and Methods

This study utilized four key aquatic model species, *D. magna*, *A. salina*, *P. tricornutum*, and *S. polyrhiza*, to represent distinct primary trophic levels and environmental compartments, thereby providing a broad overview of antibiotic impacts across aquatic ecosystems.

*D. magna* and *A. salina* (both primary consumers) were included as reference invertebrates in ecotoxicological testing, as they are commonly used to evaluate chemical effects in freshwater and saltwater ecosystems. The macrophyte *S. polyrhiza* and the diatom *P. tricornutum* were selected to represent primary producers in freshwater and marine compartments, respectively. Notably, *S. polyrhiza* was chosen over *Raphidocelis subcapitata* to represent freshwater primary producers because it offers a different level of biological organization and is known to be sensitive to fluoroquinolones, which disrupt DNA and impede photosynthesis [20]. By excluding *R. subcapitata* from our study, we also took into account that its sensitivity to CIP, SMX, and other antibiotics has been investigated in several studies [21,22]. This combination of species enables a thorough evaluation of sensitivity across primary trophic levels, involving both producers and consumers.

### 2.1. Chemicals

Analytical-grade Ciprofloxacin, CAS number: 85721-33-1 and purity ≥ 99%, and Sulfamethoxazole, CAS number: 723-46-6 and purity ≥ 98%, were supplied by Sigma-Aldrich (Milan, Italy). Stock solutions of each compound (100 mg L^−1^) were prepared by gentle stirring overnight at 37 °C in the corresponding test medium. Then, the solutions were stored in the dark at 4 °C until the start of the test. For analytical determination of compound concentrations in test media, Methanol (LC–MS grade) was purchased from Carlo Erba Reagents S.r.l., Milan, Italy; Formic acid (LC–MS grade, purity > 98.0%) and ammonium acetate (ACS reagent, purity ≥ 97%) were obtained from Merck KGaA (Darmstadt, Germany). For the test on *D. magna* and *S. polyrhiza*, a 100 mg L^−1^ stock solution of each compound under investigation was prepared in Rocchetta© water (Rocchetta S.p.A., Gualdo Tadino, Italy; pH 7.6, dry residue 181.6 mg L^−1^) or in sterile Swedish Standard (SIS) medium, respectively, as specified in OECD Test Guideline 221 [23]. For the tests on *A. salina* and *P. tricornutum*, a 100 mg L^−1^ stock solution of each compound under investigation was prepared in synthetic sea water, in accordance with ISO 10253:2024 [24]. The pH was measured with a BASIC20 pH-meter (CRISON, Carpi, Italy); when necessary, it was adjusted to 7.5 (Rocchetta© water), 6.5 (Swedish Standard (SIS) medium) and 8.0 (synthetic sea water), by adding a few drops of hydrochloric acid or sodium hydroxide solution.

### 2.2. Phaeodactylum tricornutum Culture Conditions

An axenic strain (1a) of the unicellular alga *P. tricornutum*, was obtained from the Culture Collection of Algae at the Department of Experimental Phycology, University of Göttingen, Germany. The algae were propagated in sterile synthetic seawater medium, consistent with ISO 10253:2024 [24], supplemented with vitamins B12, B8, and B1 at final concentrations of 1, 1, and 200 µg L^−1^, respectively. To maintain suspension, filtered air was continuously bubbled through the medium. Cultures were kept under constant illumination (10,000 lux) at a temperature of 20 ± 2 °C. Approximately 10 days before testing, a fresh culture was started to ensure that the algae were in the exponential phase of growth during the experimental procedures. Cell counts were performed daily using a Bürker counting chamber to monitor algal growth; inocula for each test were taken when the logarithmic increase in cell number confirmed exponential growth (doubling time ≈ 24 h). Tests were initiated with approximately 1 × 10^4^ cells mL^−1^, in line with the recommended starting density by the ISO 10253:2024 guidelines, to maintain exponential growth and prevent transition to the stationary phase over the 72-h exposure.

### 2.3. Spirodela polyrhiza Culture Conditions

*S. polyrhiza* turions were initially purchased from MicroBioTests Inc.© (Ghent, Belgium). Upon arrival, they were incubated in T-75 flasks (SARSTEDT^®^, AG & Co. KG, Nümbrecht, Germany) containing 80 mL of sterile Swedish Standard (SIS) medium at 24 ± 2 °C under fluorescent lighting (10,000 lux) for approximately 3 days to stimulate germination, as described in OECD Test Guideline 221 [23]. Then, the produced fronds were maintained in groups of seven in T-75 flasks filled with sterile SIS medium, kept at 24 ± 2 °C under a lower light intensity (6000 lux). The culture medium was refreshed weekly, and excess fronds were periodically thinned out under a laminar flow hood to prevent contamination. The *S. polyrhiza* monoculture was maintained under these controlled conditions for at least three months prior to starting the experiments.

### 2.4. Daphnia magna Culture Conditions

A stable and healthy population of *D. magna* clones has been maintained in our laboratory for several years. The original stock was sourced from a local supplier. The specific steps taken to establish and maintain this culture are detailed in an earlier publication [25]. Briefly, groups of 50 *D. magna* individuals were kept in 750 mL of Rocchetta© water, supplemented with 0.01 mg L^−1^ sodium selenite. The cultures were housed in a temperature-controlled incubator set to 20 ± 1 °C, with a 16-h (100 lux): 8-h dark photoperiod. The crustaceans were fed three times per week, just after water renewal, with *Scenedesmus dimorphus*, at concentrations of 1.1 × 10^6^ cells/mL up to 4 days of age, 1.3 × 10^6^ cells/mL between 4 and 10 days, and 1.6 × 10^6^ cells/mL thereafter. Over time, the health of the culture was confirmed by low mortality rates (≤2% per week), high reproduction rate (about 15 neonates per brood), and the absence of ephippia and/or males. The cultures were refreshed monthly. To verify the stability of the *D. magna* population, the sensitivity of the clone to potassium dichromate was monitored at least once every four months.

### 2.5. Phaeodactylum tricornutum Growth Inhibition Test

The toxicity of the two antibiotics on the unicellular marine alga *P. tricornutum* was evaluated using a modified version of the ISO 10253:2024 guideline—Marine Algal Growth Inhibition Test [24]. Modifications to the standard protocol included a reduction in vessel and solution volumes: instead of 250 mL Erlenmeyer flasks with 100 mL of medium, flat-bottom P6 plates (SARSTEDT^®^) were used, each containing 10 mL of test solution. The experimental setup utilized sterile synthetic seawater as the medium, and the light and temperature conditions matched those of the algal culture environment. All experiments were conducted aseptically and in triplicate [26]. A negative control group—comprising six biological replicates with pure synthetic seawater—was included in each assay. The assayed concentrations of CIP were 100, 50, 25, 12.5, 6.25, and 3.13 mg L^−1^, whilst those of SMX were 15, 5, 1.67, 0.56 and 0.19 mg L^−1^. After 72-h exposure, algal cell concentrations were determined using a Burker counting chamber and a Keyence VHX-X1-S770E digital microscope (Keyence Corporation, Osaka, Japan). To assess whether the test compounds had algistatic or algicidal properties, a 50 μL aliquot was taken from the concentration that caused the strongest inhibition of algal growth. The aliquot was transferred to fresh synthetic seawater (10 mL) and maintained under standard culture conditions for an additional 9 days. If regrowth occurred, the effect was classified as algistatic; if no regrowth was observed, it was considered algicidal [27]. Since sulphonamides, like SMX, work by inhibiting folate synthesis, it was hypothesized that adding folic acid to the medium might reduce or reverse the growth-inhibiting effects of SMX.

Therefore, a further test was conducted, exposing *P. tricornutum* to the EC_50_ of SMX, in the presence of 100 ng L^−1^ Folic Acid. The concentration of folic acid to be used was established on the basis of a similar experiment conducted previously on the freshwater green alga *R. subcapitata* [28].

### 2.6. Spirodela polyrhiza Growth Inhibition Test

Toxicity assessment in the greater duckweed *S. polyrhiza* was carried out in general accordance with the OECD 221 protocol—*Lemna* sp. Growth Inhibition Test [23]. The modifications included: (a) the use of a duckweed of the genus *Spirodela* instead of *Lemna*; (b) the use of T-75 cell culture flasks (SARSTEDT^®^) as test vessels, each filled with 80 mL of either test solution or control medium; and (c) the evaluation of multiple endpoints, specifically: frond number, total frond area, root number, average root length, fresh weight and frond colouration. The experiments were conducted under sterile conditions using the Swedish Standard (SIS) medium, with temperature and light settings identical to those used for routine culturing. All tests were performed in triplicate. A negative control group, consisting of three biological replicates in pure SIS medium, was included in each assay. The tested concentrations of SMX and CIP were 2, 0.62, 0.19, 0.06, 0.02 mg L^−1^. After a 7-d exposure period, fronds and roots were photographed using a Nikon D3400 camera (Nikon Corporation, Tokyo, Japan), and the following parameters were measured using ImageJ^®^ software, version 1.53k (National Institutes of Health, Bethesda, MD, USA): number of fronds, total frond area, number of roots, average root length, and colony greenness. In particular, the greenness of each colony was measured as the ratio between the average G component and the sum of the average R, G and B components [29]. At the end of the test, for fresh weight determination, the plants from each flask were collected, dried with blotting paper and then centrifuged (10’, 3000 RPM) at room temperature in 50 mL tubes containing blotting paper at the bottom. The fronds were then transferred to Eppendorf tubes of known tare, and weighed.

### 2.7. Artemia salina Acute Immobilisation Test with 10-d Follow-Up

*A. salina* cysts were initially obtained from a reputable local supplier. Their high quality was confirmed by a hatching success rate exceeding 90%. Seventy-two hours before the acute toxicity tests, the cysts were incubated in synthetic seawater [24] at 24 ± 2 °C under fluorescent light (10,000 lux) to obtain viable nauplii. Given the absence of a standardized protocol for toxicity testing with Artemia, the experimental procedure aligned with the OECD Guideline 202—*Daphnia* spp., Acute Immobilisation Test [30], supplemented with recommendations from Libralato et al. [31]. This assay evaluates the organisms’ survival following 48 h of exposure. For each antibiotic, five concentrations were prepared in a geometric series with a dilution factor of 2 (100, 50, 25, 12.5, and 6.25 mg L^−1^). Only *A. salina* nauplii at developmental stages between instar II and III were used for the test. Acute exposure was conducted in 24-well plates (SARSTEDT^®^), with 30 individuals per treatment group. After brief rinsing in their respective test solutions, the nauplii were transferred (10 per well) into three wells, each containing 1 mL of test or control solution (synthetic seawater). Incubation was performed in complete darkness at 25 ± 2 °C. Immobilisation was assessed at both 24 and 48 h. Before the test began, a reference group of 30 neonates (not intended for the test) was fixed in 100% ethanol and photographed using a Keyence VHX-X1-S770E digital microscope. Body length (from eye to tail) was measured using the VHX Measurement Data Tabulation Tool software version 3.3.19.528(Keyence Corporation, Osaka, Japan) to establish the average baseline size at time zero.

When unusual swimming behaviour was observed at the end of the acute test, a potential delayed toxicity effect was further assessed. For this purpose, a 10-d post-exposure observation in clean medium was added after the standard 48-h test, during which lethality and daily growth were monitored. For this purpose, at the end of the exposure period, surviving individuals were transferred—maintaining the identity of the treatment—into individual wells of P-6 plates (SARSTEDT^®^) filled with 10 mL of fresh synthetic seawater. Every other day, dead individuals were counted and removed, the medium was renewed, and the organisms were fed with *P. tricornutum* at a concentration of 5.3 × 10^5^ cells mL^−1^. To calculate growth rates, body length was measured for each surviving individual at the end of the 10-day follow-up. The daily growth rate was then determined by subtracting the average baseline length at time zero from each organism’s final body length and dividing the resulting value by the total experimental duration (12 days).

### 2.8. Daphnia magna Acute Immobilisation Test with 10-d Follow-Up

Acute toxicity testing was carried out following the OECD Guideline 202, *Daphnia* spp., Acute Immobilisation Test [30], which evaluates the number of immobilised individuals after 24 and 48-h exposure. For each antibiotic tested, five concentrations were prepared in a geometric progression with a dilution factor of 2. Specifically, the concentrations of CIP were 50, 25, 12.5, 6.25 and 3.13 mg L^−1^, whilst those of SMX were one order of magnitude higher (500, 250, 125, 62.5, and 31.25 mg L^−1^). Only third to fifth brood neonates aged between 12 and 24 h were used for the tests. They were collected 12 h before the beginning of the test, transferred to a 250 mL beaker with 200 mL of fresh medium, and fed with *S. dimorphus* at 1.1 × 10^6^ cells mL^−1^. Although pre-feeding is not specified in the OECD guideline, previous experience has shown it enhances survival in control groups and is therefore advisable [14]. Acute exposure tests were conducted using the 30-well plates provided in the Daphtoxkit F© (MICROBIOTEST, Ghent, Belgium). In alignment with OECD 202 specifications, each treatment group included 20 daphnids. After brief rinsing in their respective solutions, individuals were distributed (five per well) into four wells, each containing 10 mL of either the test solution or the control medium (Rocchetta© water). Temperature and light conditions matched those used in culture maintenance. As indicated by the guideline, after 24 and 48 h the number of immobilised individuals was recorded. Furthermore, to investigate potential delayed effects, an additional 10-d observation period in pure medium was implemented. For this purpose, surviving individuals were transferred, keeping track of their original treatment group, into individual beakers with 50 mL of pure Rocchetta© water. Then, every other day, immobilised individuals were counted and removed, the medium was renewed, and the crustaceans were fed with *S. dimorphus* (see Section 2.4). Another endpoint considered after the follow-up was the growth rate. Therefore, before the start of the standard 48 h test, 30 neonates (not intended for the test) were preserved in 100% ethanol and photographed under an Olympus CX41 RF microscope equipped with a Nikon D3400 camera. Individual body length (measured from the eye to the base of the tail spine) was determined using ImageJ© software to establish the average baseline length at time zero. The same procedure was applied at the end of the 10-day follow-up for all surviving daphnids. The daily growth rate was calculated for each surviving individual by subtracting the average baseline length at time zero from its body length measured after the 10-day follow-up and dividing the resulting value by the total experimental duration (12 days).

### 2.9. Artemia salina Cysts Hatching Test

For the embryo hatching assay, *A. salina* cysts were used. The experimental setup followed an approach that we had previously validated in *D. magna* [14,15,32], thereby ensuring methodological consistency and interspecies comparisons. The test was conducted in 24-well plates (SARSTEDT^®^), with 30 cysts assigned to each treatment group. Each antibiotic was tested at five concentrations arranged in a geometric series with a dilution factor of 2 (100, 50, 25, 12.5, and 6.25 mg L^−1^). Cysts were pre-incubated in pristine seawater for 4 h to allow proper rehydration. Following this step, and after a brief rinse in their respective test solutions, the cysts (n = 30) were randomly distributed (10 per well) into three wells, each containing 1 mL of either test solution or control medium (synthetic seawater). Incubation was carried out over a period of 3 days under standard conditions for *A. salina* hatching, including controlled light and temperature settings (as described above for the preparation of the acute toxicity test). The number of hatched embryos was recorded at 24-h intervals. Upon completion of the test, all hatched individuals from both exposed and control groups were preserved in pure ethanol for imaging, growth analysis, and malformation assessment.

### 2.10. Daphnia magna Embryonic Development Test

Given that previous studies had demonstrated embryotoxic effects of the antibiotics Flumequine, Enrofloxacin, and Levofloxacin in *D. magna* [14,15,32], we extended the investigation to assess the potential embryonic toxicity of CIP and SMX. Since standardized protocols for *D. magna* embryo or cyst hatching assays are missing, our experimental setup followed previously validated approaches in our lab [14,15,32]. In brief, early-stage embryos (stage 1) were carefully collected from gravid females. This was achieved by gently restraining the adult’s head with a dissecting probe whilst a second probe was used to separate the carapace and release the embryos [33]. Each antibiotic was tested at five concentrations arranged in a geometric series with a dilution factor of 2 (100, 50, 25, 12.5, and 6.25 mg L^−1^). Embryos were randomly assigned to treatment groups, briefly rinsed in the corresponding test solution, and transferred—three per well—into 24-well plates (SARSTEDT^®^) containing 1 mL of either test solution or pure Rocchetta© water as a negative control (n = 15). The plates were incubated under standard light and temperature conditions, as already indicated for *D. magna* culturing. Embryo hatching was monitored daily over a 3-d period. After incubation, all neonates—both from treated and control groups—were preserved in absolute ethanol for subsequent imaging, growth measurement, and morphological assessment.

### 2.11. Chemical Analysis

At the beginning and end of each test, samples of freshly prepared and spent solutions were taken from the highest and lowest assayed concentrations to evaluate the stability of the two antibiotics. All analyses were performed by liquid chromatography with mass spectrometry detection (LC–MS/MS). The chromatographic separation was achieved using an Accela 600 HPLC pump with CTC automatic injector (Thermo Fischer Scientific, Waltham, MA, USA) equipped with a C18 Kinetex (100 × 2.1 mm, 2.6 μm) analytical column by Phenomenex (Torrance, CA, USA). Chromatographic separation was carried out using a mobile phase consisting of ammonium acetate 0.01 M, pH 2.5 with 0.1% formic acid (eluent A) and Methanol with 0.1% formic acid (eluent B). Optimized separation was obtained using the following gradient program (%A:%B): 80:20 at 0 min, 10:90 at 4 min, held at 10:90 until 6 min, 80:20 at 7 min, held at 80:20 until 9 min to re-equilibrate the system. The flow rate was set at 0.20 mL min^−1^, and the injection volume was 10 μL. The mass detection was achieved with an LTQ XL ion trap mass spectrometer (Thermo Fischer Scientific, San Jose, CA, USA), equipped with a heated electrospray ionization (HESI-II) probe. The mass spectra were acquired using positive-ion ESI mode. The two precursor ions (MH+) considered for ciprofloxacin and sulfamethoxazole were 332 and 254, respectively. The diagnostic fragments that were monitored for ciprofloxacin (MS3) and sulfamethoxazole (MS2) were *m*/*z* 245 and 268 and *m*/*z* 156 and 188, respectively. The linearity of response was verified in the 2.5–200 ng/mL range. In order to match this concentration range, samples were accordingly diluted in mobile phase before injection, when necessary.

### 2.12. Data Analysis

All statistical analyses were performed using GraphPad Prism version 8.4.3. When data consistently meet assumptions of normality, the One-way Anova test was applied, followed by Dunnett’s test, otherwise the non-parametric Kruskal–Wallis test, followed by Dunn’s test, was preferred. Normality of the data was assessed through the Shapiro–Wilk test. *p*-values were corrected for multiple comparisons, and statistical significance was set at *p* ≤ 0.05. For *D. magna* immobilisation, *A. salina* survival, and *S. polyrhiza* and *P. tricornutum* growth inhibition, data were modelled using a four-parameter logistic dose–response curve [34]. Accordingly, median effective concentration (EC_50_) values and their corresponding confidence intervals (CI) were calculated.

## 3. Results

Both antibiotics proved to be fairly stable under the various experimental conditions applied (light, temperature, and incubation time). Measured concentrations in freshly prepared test solutions were within 89–105% and 96–109% of the nominal values for CIP and SMX, respectively. Degradation rates, calculated by comparing peaks of fresh and spent solutions, were always below 8% for CIP and below 15% for SMX. Since the concentrations of both compounds remained within ±20% of the nominal values, test results were expressed on a nominal basis.

**Figure 1:**
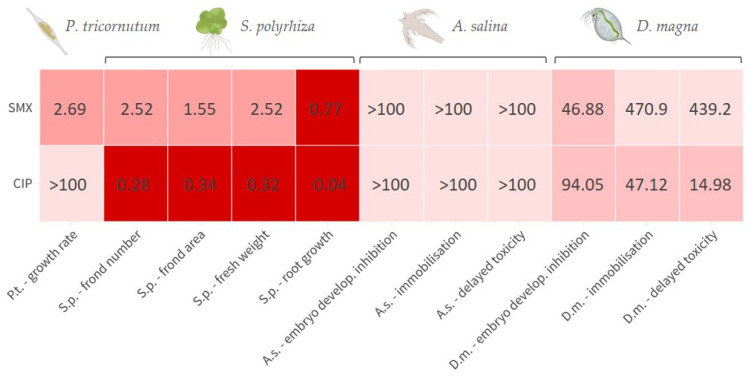
Heatmap reporting the estimated EC_50_ values (mg L^−1^) for sulfamethoxazole (SMX) and ciprofloxacin in each test in the four model organisms: *P. tricornutum* (*P.t.*), *S. polyrhiza* (*S.p.*), *A. salina* (*A.s.*), *D. magna* (*D.m.*). The colour gradient reflects the EC_50_ value, ranging from low (red, very toxic) to high (pinkish white, non-toxic), with intermediate shades indicating toxic (pale red) and harmful (pink) levels, according to the OECD harmonised system [35]. Created with BioRender.com and Flourish.

**Figure 2:**
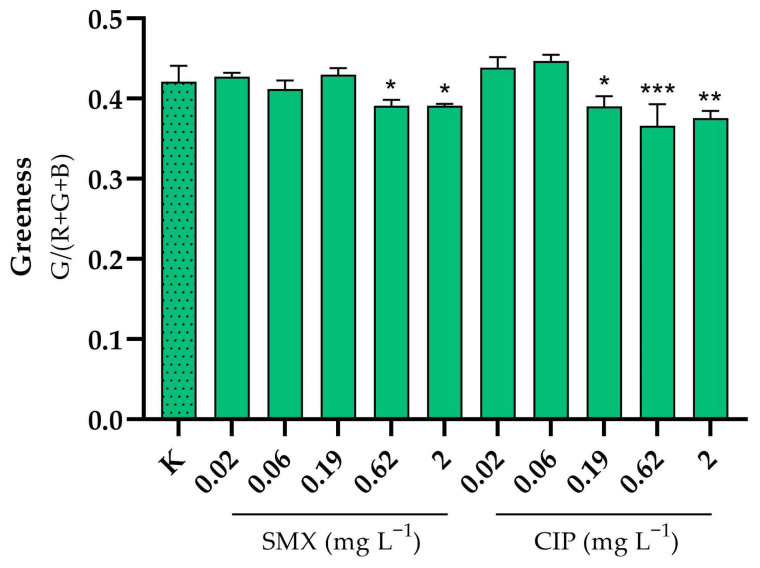
Greenness of *Spirodela polyrhiza* after 7 days of exposure to sulfamethoxazole (SMX) and ciprofloxacin (CIP). Colony greenness is the ratio between the average green (G) component and the sum of the average red (R), green (G) and blue (B) components [29]. K = control. Error bars show standard error. * Significantly different from the control (*p* < 0.05); ** significantly different from the control (*p* < 0.01); *** significantly different from the control (*p* < 0.001); one-way Anova followed by Dunnett’s post hoc test.

**Figure 3:**
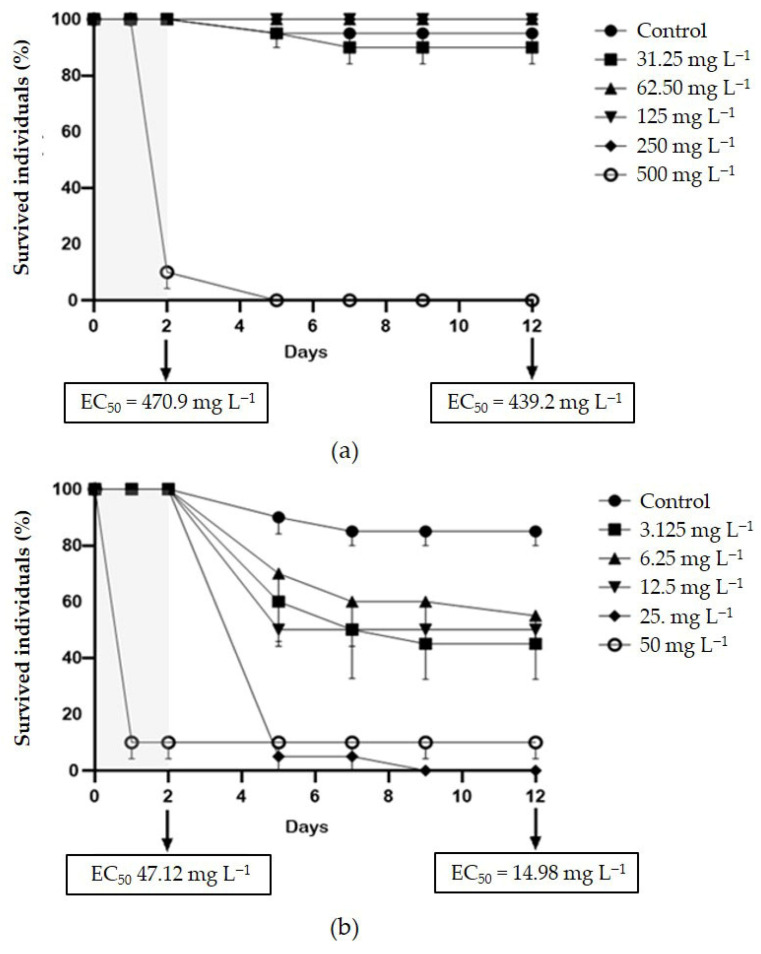
Survival curves of *D. magna* over 48-h exposure to (**a**) sulfamethoxazole (SMX) and (**b**) ciprofloxacin (CIP), with 10-d follow-up in pure medium. Error bars show standard error.

**Figure 4:**
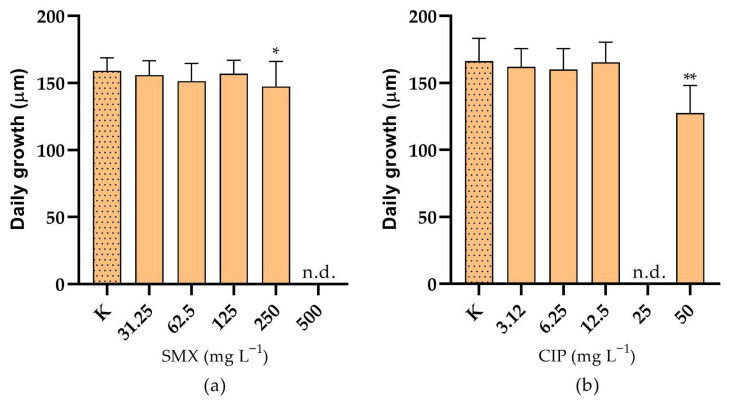
Daily growth of *D. magna* over 12 days, i.e., 2 days of exposure to (**a**) sulfamethoxazole (SMX) and (**b**) ciprofloxacin (CIP), plus an additional 10-d follow-up in pure medium. K = control; n.d. = no data available (almost all the exposed animals died during the experiment). Error bars show standard error. * Significantly different from the control (*p* < 0.05); ** significantly different from the control (*p* < 0.01); one-way Anova followed by Dunnett’s post hoc test.

For the convenience of the reader, the results of the various tests are reported in Supplementary Materials (Table S1, Figure S1), and summarised in a heatmap (Figure 1); toxicity categories were assigned as described in the OECD harmonised system [35]. The growth rate inhibition EC_50_ of SMX on *P. tricornutum* was 2.69 (1.82–4.03) mg L^−1^. The effect was algistatic. It was also verified that when the alga was exposed to this concentration in the presence of 100 ng L^−1^ folic acid, no attenuation of toxicity occurred, as a growth inhibition of approximately 50% was observed after 72 h. At the assayed concentrations, CIP did not affect *P. tricornutum* growth. Depending on the endpoint considered, the EC_50s_ of SMX on *S. polyrhiza* were in the range 0.77–2.52 mg L^−1^, whilst those of CIP were in the range 0.04–0.34 mg L^−1^. Particularly with CIP, there was also some significant effect on greenness at the two highest doses tested (Figure 2). The brine shrimp *A. salina* proved to be very resistant to SMX and CIP toxicity, with no significant acute effects on neonates, even after the 10-d follow-up, and also not in embryos (Figure S1). However, it is important to note the occurrence of certain physical malformations, although not lethal within the 72-h experimental period, in embryos exposed to CIP and SMX. These abnormalities included the absence of the ocellus, missing limbs, and other body deformities (Figure S2). Notably, such malformations were never observed in organisms developed in pure medium. *D. magna* embryos were instead moderately sensitive to the toxicity of SMX and CIP, with EC_50s_ for development inhibition of 41.27 and 95.93 mg L^−1^, respectively. In addition, physical malformations have been observed in embryos exposed to CIP and SMX, which, although not lethal within the 72-h experimental period, could potentially exacerbate the toxic effects of these substances over longer exposure times. The abnormalities, never reported in the control group, included the absence or deformation of the ocellus, incomplete development, and other structural deformities (Figure S3). Delayed acute toxicity of SMX was not reported in *D. magna* juveniles, with similar and very high EC_50_ values after 48 h and 10 d of follow-up: 470.9 and 439.2, respectively (Figure 3a). On the contrary, CIP showed a remarkable delayed toxicity, with an EC_50_ of 47.12 mg L^−1^ after 48 h, which dropped to 14.98 mg L^−1^ after 10-d follow-up (Figure 3b). Moreover, a slight reduction in *D. magna* daily growth was observed following exposure to both CIP (50 mg L^−1^) and SMX (250 mg L^−1^) (Figure 4).

## 4. Discussion

According to the review by Zhou et al., sulphonamides are toxic to algae, with EC_50s_ in the range 1.54–32.25 mg L^−1^ [36]. The toxicity of SMX for *P. tricornutum* (EC_50_ of 2.69 mg L^−1^) reported here is in good agreement with that recently indicated by Feng and colleagues [7] after 96-h exposure (EC_50_ = 4.65 mg L^−1^) and, together with data reported by De Orte et al., for sulfadiazine (EC_50_ = 0.11 mg L^−1^), confirms the high sensitivity of this marine diatom to sulphonamides [37]. Conversely, the fluoroquinolone CIP showed no toxicity to the marine algae. As for SMX, the addition of folic acid to the culture medium did not limit its toxicity towards *P. tricornutum*. This indicates that the antibiotic’s inhibition of algal growth may not be due to the inhibition of folate synthesis, but rather to a different mechanism that is distinct from the one responsible for its antimicrobial activity. However, it is also possible that the dose of folic acid added (100 ng L^−1^) was not sufficient. It is important to highlight that a similar experiment conducted on the freshwater green alga *R. subcapitata* found that the same concentration of the vitamin could completely reverse the growth inhibition caused by 2.19 mg L^−1^ of sulfadiazine [28]. Further experiments using higher concentrations of folic acid may clarify these aspects.

Of the several endpoints considered when testing the two antibiotics on *S. polyrhiza*, root growth was the most sensitive, with a calculated EC_50_ of 0.77 and 0.04 mg L^−1^ for SMX and CIP, respectively. These results confirm their high toxicity to aquatic plants [38,39]. Root growth in the case of *S. polyrhiza* can be a very sensitive endpoint because of the considerable number of roots that this species, unlike *Lemna* spp., is capable of developing. However, for CIP, all four evaluated endpoints resulted in EC_50_ values ≤ 0.34 mg L^−1^, highlighting a considerable level of toxicity. This finding is particularly concerning, as floating macrophytes play a vital role in sustaining the health, balance, and ecological functionality of freshwater ecosystems [40]. Furthermore, floating plants offer numerous ecological benefits to fish, crustaceans, and other aquatic organisms by providing oxygen, food, habitat, and resources, as well as shelter from predators and suitable sites for spawning and juvenile development [41,42]. Consequently, the environmental depletion of these species resulting from CIP contamination could trigger cascading effects throughout the freshwater ecosystem, potentially disrupting trophic interactions and compromising overall ecosystem functionality.

No significant effects were found when testing the two antibiotics on the saltwater crustacean *A. salina*. Indeed, despite the numerous advantages offered by this species for ecotoxicity tests, i.e., year-round availability without the need for culturing, rapid hatching from durable commercial cysts, population homogeneity, and cost-effectiveness [31], its sensitivity to toxic substances appears to be generally lower than that of *Daphnia* spp. [43,44,45], at least when considering the classic endpoints. *D. magna*, instead, was sensitive to both antibiotics. Notably, embryos were more sensitive to SMX, whilst juveniles were more sensitive to CIP. To our knowledge, the toxicity of sulphonamides has never been tested on *D. magna* embryos; however, in a previous reproduction test conducted with sulfamethazine on *D. magna*, a strong reproduction inhibition (EC_50_ = 4.25 mg L^−1^) was observed, which was consistent with the observation of apparently regular reproduction cycles ending in the abortion of numerous eggs [17]. The embryotoxicity observed in *D. magna* following exposure to sulphonamides can be mechanistically explained by their inhibition of folic acid synthesis. Sulphonamides competitively inhibit dihydropteroate synthase, a key enzyme in the folate pathway, thereby disrupting the synthesis of purine and pyrimidine nucleotides essential for DNA replication. Since embryonic development involves rapid and continuous cell division, this disruption disproportionately affects embryonic cells, leading to arrested development and egg abortion. In essence, this mode of action makes the early stages of development particularly vulnerable to exposure to sulphonamides. On the other hand, the toxicity of CIP to *D. magna* juveniles that had been exposed to the drug only during their neonatal life, confirms the ability of fluoroquinolones to exert delayed toxicity after the 48-h acute exposure envisaged by the standard immobilisation test [14]. Indeed, the EC_50_ calculated after the 10-d follow-up in pure medium (14.98 mg L^−1^), was more than three times lower than that calculated after the 48-h exposure to CIP (47.12 mg L^−1^). This result once again highlights the limitations of the acute toxicity test, and suggests that conducting a follow-up in a clean medium could make this standard assay considerably more informative [46]. In contrast, the delayed toxicity of SMX was found to be negligible as the 48-h EC_50_ (470.9 mg L^−1^) was very close to that obtained after follow-up (439.2 mg L^−1^).

**Figure 5:**
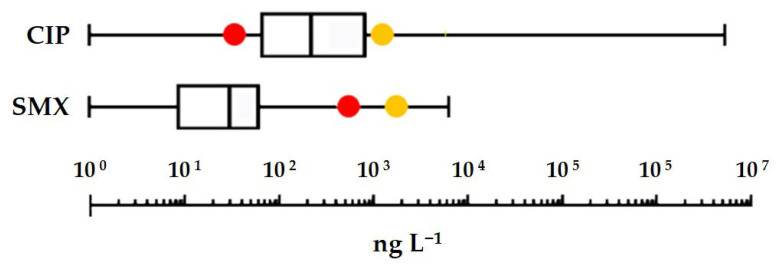
Box plot of the maximum concentrations reported per study in freshwater environments for ciprofloxacin (CIP) and sulfamethoxazole (SMX) [12]. Red dots are the Predicted No Effect Concentration (PNEC) values obtained from the current experimentation; yellow dots are the PNEC values previously obtained from Species Sensitivity Distribution curves.

In general terms, the toxicity of the two antibiotics does not differ significantly from that of the classes of compounds to which they belong. Among the model organisms on which ecotoxicological assays were performed, the only one strongly sensitive to both drugs was the greater duckweed *S. polyrhiza*. It can therefore be used as a reference for risk assessment. Applying an assessment factor of 1000 to the EC_50_ measured for the most sensitive endpoint (inhibition of root growth) yields a freshwater PNEC of 0.77 and 0.04 µg L^−1^ for SMX and CIP, respectively. In Figure 5, these PNECs and those previously obtained from Species Sensitivity Distribution curves [12,47] are compared to the box plots of the highest concentrations of the two antibiotics detected in surface waters [4]. Notably, environmental concentrations considered in this comparison covered a wide geographical range (African, Asian, European, and American continents) and an extensive time period (approximately from 2006 to 2020). The comparison shows that a risk to the freshwater environment cannot be ruled out, particularly in the case of CIP, where even the least stringent PNEC, obtained from the SSD curve, is exceeded in nearly 25% of the waters analysed. Moreover, on a global scale, CIP would have a frighteningly high GWF (Grey Water Footprint): 1900 m^3^·yr^−1^ on a per capita basis [48]. And this value is probably understated as the CIP environmental load coming from the metabolism of enrofloxacin, a largely used veterinary fluoroquinolone, was not originally included in the calculation [49].

## 5. Conclusions

This study provides new insights into the aquatic toxicity of CIP and SMX, focusing on primary producers and primary consumers. Among the organisms tested, the freshwater macrophyte *S. polyrhiza* emerged as the most sensitive species, with EC_50_ values in the sub-mg L^−1^ range, particularly for root growth inhibition. These findings reinforce the role of aquatic plants as critical sentinels for antibiotic pollution, given their ecological importance for habitat provision, nutrient cycling, and food-web stability [40,41]. In contrast, the marine alga *P. tricornutum* displayed significant sensitivity to SMX but not to CIP, whereas the brine shrimp *A. salina* was largely insensitive to both compounds. Lastly, *D. magna* showed intermediate sensitivity, with embryos affected by SMX and juveniles by CIP, the latter also exhibiting delayed toxicity beyond the standard acute test period. This underscores the importance of including extended post-exposure observations to capture sublethal and latent effects that would otherwise remain undetected.

From a regulatory perspective, the PNECs derived from *S. polyrhiza* data (0.77 µg L^−1^ for SMX and 0.04 µg L^−1^ for CIP) are exceeded by reported environmental concentrations, especially for CIP. This points to a tangible ecological risk in freshwater systems, where communities of aquatic macrophytes function as keystone components, and their impairment could cascade across trophic levels.

Overall, the results confirm that fluoroquinolones and sulphonamides, despite their distinct modes of action, can both impair early life stages of invertebrates and compromise the growth of aquatic primary producers. Given their widespread use and persistence in the environment, the ecological footprint of these antibiotics should not be underestimated. Future risk assessments would benefit from (i) considering sensitive endpoints such as root growth in macrophytes, (ii) extending acute toxicity protocols with post-exposure follow-ups, and (iii) integrating mixture toxicity and chronic exposure scenarios to better reflect environmental conditions.

## Data Availability

The raw data supporting the conclusions of this article will be made available by the authors on request.

## Supplementary Materials

The following supporting information can be downloaded at: https://www.mdpi.com/article/10.3390/toxics13110979/s1, Figure S1: Effects of CIP and SMX on *A. salina* and *D. magna* growth rate, hatching rate and embryos development; Figure S2: Examples of living *A. salina* embryos observed at the end of the 72-h hatching test; Figure S3. Examples of living *D. magna* embryos observed at the end of the 72-h hatching test; Table S1: Summary of the test results.

## Abbreviations

The following abbreviations are used in this manuscript:

CIP	Ciprofloxacin
SMX	Sulfamethoxazole
EC_50_	Median Effective Concentration
PNEC	Predicted No-Effect Concentration
SSD	Species Sensitivity Distribution
