# Supplementary material for: Aquatic Toxicity of Antibiotics Ciprofloxacin and Sulfamethoxazole: Significant Impact on Early Life Stages of Crustaceans, a Marine Diatom, and a Freshwater Plant
Source: Toxics. 2025 Nov 14;13(11):979. doi: 10.3390/toxics13110979 (PMC12656477; doi:10.3390/toxics13110979)
Supplement: Supplementary file 1 [file toxics-13-00979-s001.zip › toxics-3944369-supplementary.pdf]

Table S1. *Summary of the test results*

Model organism	***Antibiotic***	***Endpoint***	***mg L^{-1} EC_{50} (95% C.I.)***	***Toxicity****
P. tricornutum	SMX	Growth-Rate inhibition	2.69 (1.82-4.03)	Toxic**
	CIP	Growth-Rate inhibition	>100	Non-toxic
S. polyrhiza	SMX	Frond number	2.52 (1.87-3.17)	Toxic
	CIP	Frond number	0.28 (0.19-0.37)	Very toxic
	SMX	Frond area	1.55 (1.26-1.83)	Toxic
	CIP	Frond area	0.34 (0.22-0.45)	Very toxic
	SMX	Fresh weight	2.52 (1.91-3.08)	Toxic
	CIP	Fresh weight	0.32 (0.24-0.40)	Very toxic
	SMX	Root growth	0.77 (0.61-0.92)	Very toxic
	CIP	Root growth	0.04 (0.02-0.05)	Very toxic
A. salina	SMX	Embryo development inhibition	>100	Non-toxic
	CIP	Embryo development inhibition	>100	Non-toxic
	SMX	Acute immobilisation (48-h)	>100	Non-toxic
	CIP	Acute immobilisation (48-h)	>100	Non-toxic
	SMX	Delayed acute toxicity (10-d)	>100	Non-toxic
	CIP	Delayed acute toxicity (10-d)	>100	Non-toxic
D. magna	SMX	Embryo development inhibition	~46.88 (n.d.)	Harmful
	CIP	Embryo development inhibition	94.05 (83.61-116.5)	Harmful
	SMX	Acute immobilisation (48-h)	470.9 (n.d.)	Non-toxic
	CIP	Acute immobilisation (48-h)	47.12 (n.d.)	Harmful
	SMX	Delayed acute toxicity (10-d)	~439.2 (n.d.)	Non-toxic
	CIP	Delayed acute toxicity (10-d)	14.98 (n.d.)	Harmful

* based on EC_{50}, according to ENV/JM/MONO(2001)6;

** algistatic effect, not attenuated by folic acid addition (100 ng L^{-1}).

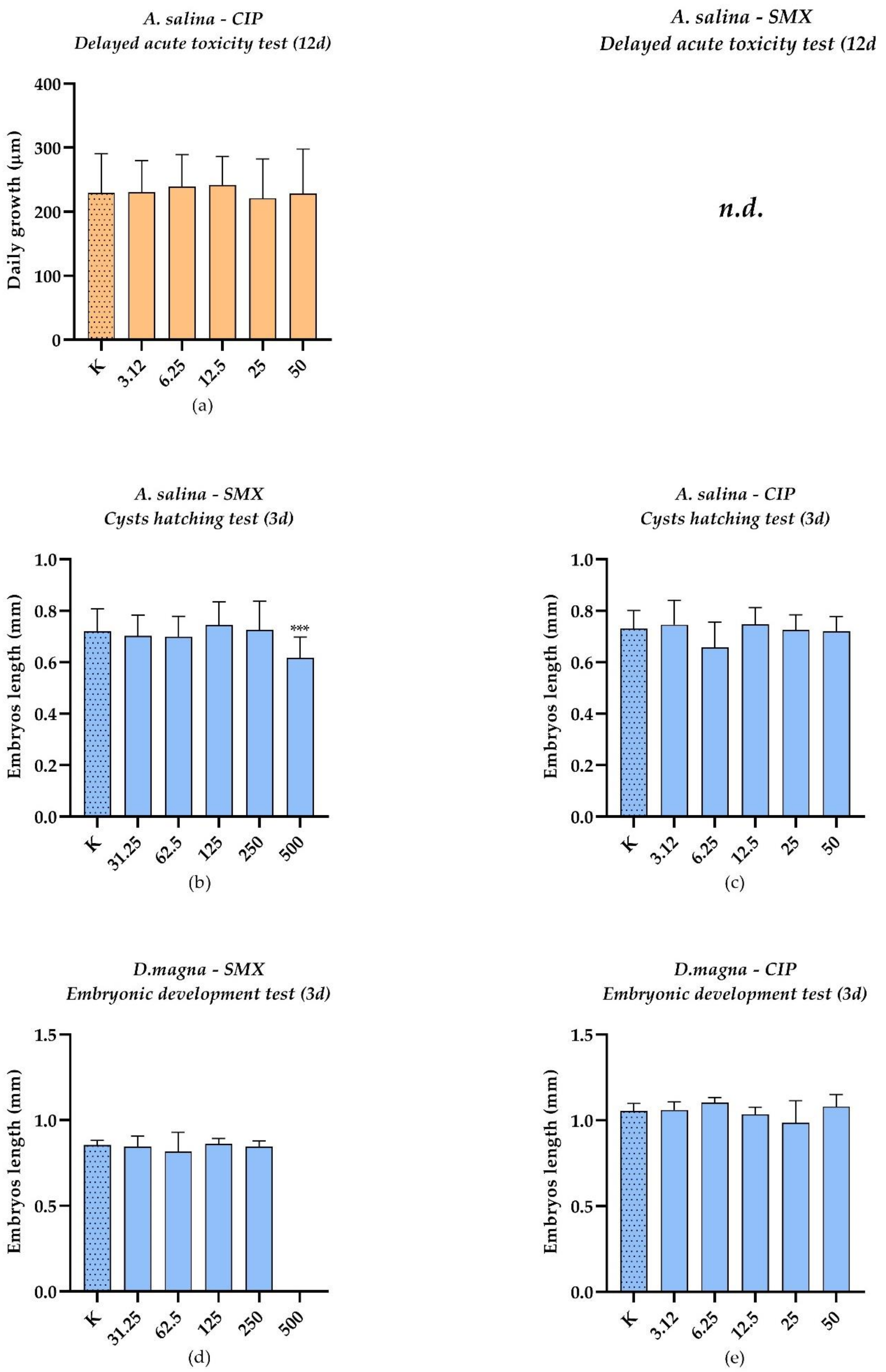

Figure S1. Effects of CIP and SMX on A. salina and D. magna growth rate, hatching rate and embryos development. *Artemia salina growth rate expressed as average daily growth during the 10-day follow-up after the acute exposure (48 hours) to CIP* ***(a)****. Effect of SMX on A. salina cyst hatching and development following exposure (72 hours).*

(**b**). *Effect of CIP on A. salina cyst hatching and development following exposure (72 hours)* (**c**). *Effect of SMX on D. magna embryo hatching and development following exposure (72 hours)* **(d)**. *Effect of CIP on D. magna embryo hatching and development following exposure (72 hours)* **(e)**. *Concentrations are expressed in mg L^{-1}. Embryonic development is expressed as total length at the end of the exposure period; SMX=sulfamethoxazole; CIP=ciprofloxacin; K=control; n.d.=no data available. Error bars show standard error. *** $p<0.001$, significantly different from the control; Kruskall-Wallis test followed by Dunn's post hoc test.*

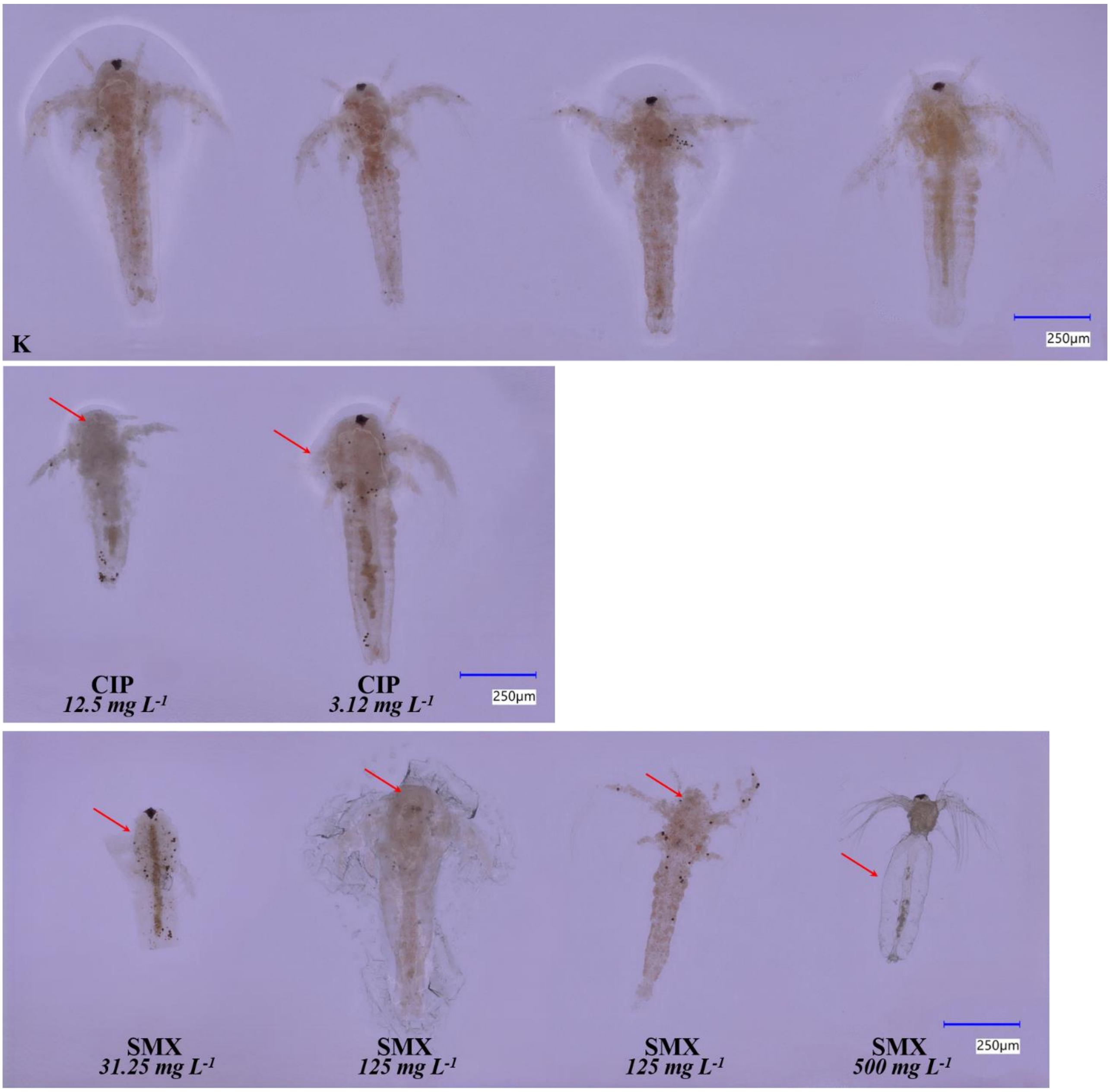

Figure S2. Examples of living A. salina embryos observed at the end of the 72-hour hatching test. *The figure compares individuals developed in a pure medium (K=control) with those exposed to ciprofloxacin (CIP) and sulfamethoxazole (SMX).*

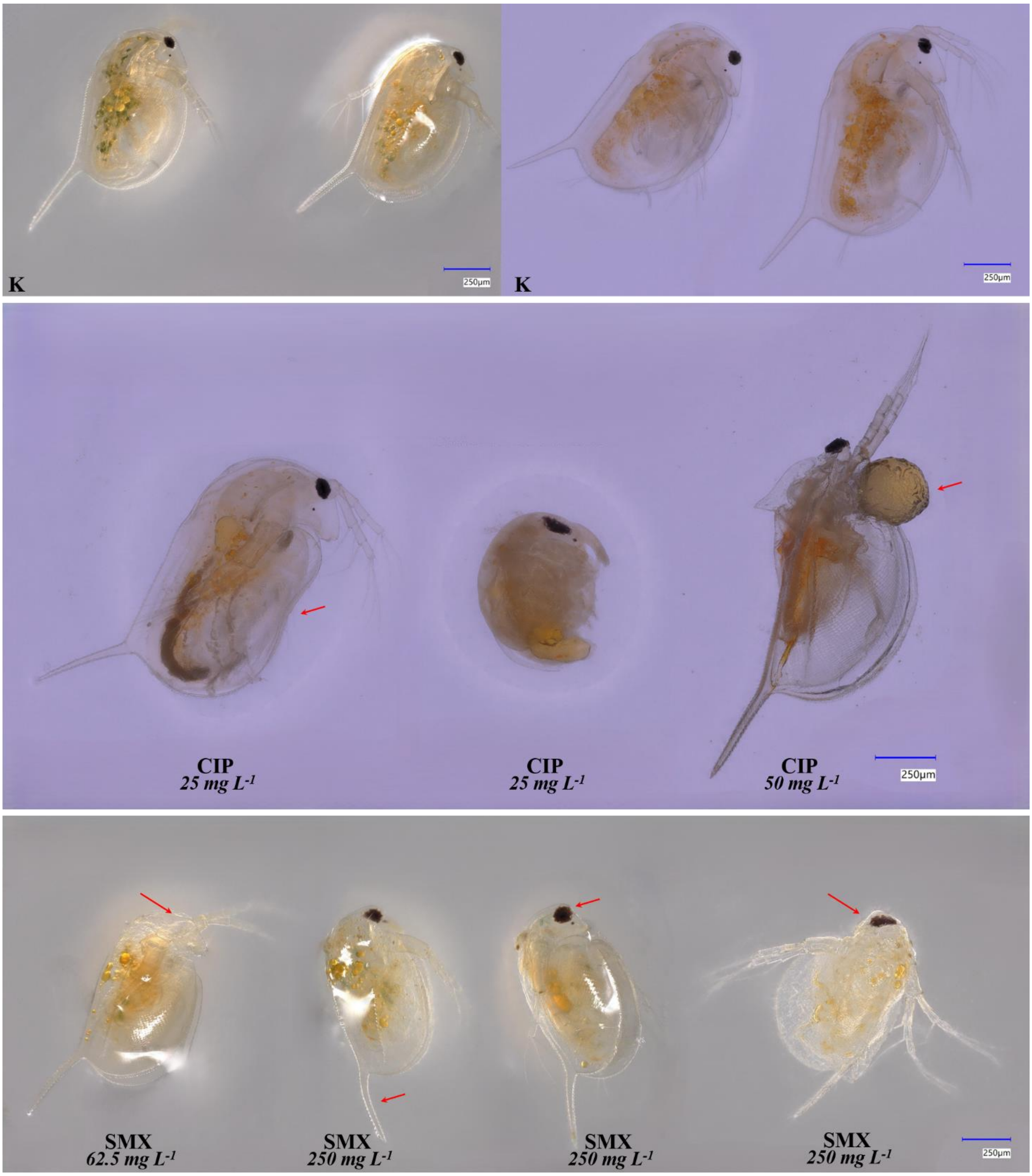

Figure S3. Examples of living D. magna embryos observed at the end of the 72-hour hatching test. *The figure compares individuals developed in a pure medium (K=control) with those exposed to ciprofloxacin (CIP) and sulfamethoxazole (SMX).*
